# *Candida auris* Direct Detection from Surveillance Swabs, Blood, and Urine Using a Laboratory-Developed PCR Method

**DOI:** 10.3390/jof6040224

**Published:** 2020-10-15

**Authors:** Robert C. Walchak, Seanne P. Buckwalter, Nicole M. Zinsmaster, Katrina M. Henn, Katelyn M. Johnson, Jolene M. Koelsch, Senait A. Herring, Lory K. Steinmetz, Katelyn A. Reed, Jean E. Barth, Jenna M. Rasmusson, Jill L. Fischer, Paula Snippes Vagnone, Priya Sampathkumar, Nancy L. Wengenack

**Affiliations:** 1 Division of Clinical Microbiology, Mayo Clinic, Rochester, MN 55902, USA; walchak.robert@mayo.edu (R.C.W.); buckwalter.seanne@mayo.edu (S.P.B.); zinsmaster.nicole@mayo.edu (N.M.Z.); henn.katrina@mayo.edu (K.M.H.); johnson.katelyn1@mayo.edu (K.M.J.); koelsch.jolene@mayo.edu (J.M.K.); herring.senait@mayo.edu (S.A.H.); steinmetz.lory@mayo.edu (L.K.S.); 2 Research and Innovation Office, Mayo Clinic, Rochester, MN 55902, USA; reed.katelyn@mayo.edu; 3 Infection Prevention and Control, Mayo Clinic, Rochester, MN 55902, USA; barth.jean@mayo.edu (J.E.B.); rasmusson.jenna@mayo.edu (J.M.R.); sampathkumar.priya@mayo.edu (P.S.); 4 Public Health Laboratory, Minnesota Department of Health, St. Paul, MN 55164, USA; jill.fischer@state.mn.us (J.L.F.); paula.snippes@state.mn.us (P.S.V.)

**Keywords:** *Candida auris*, PCR, surveillance, yeast, identification

## Abstract

*Candida auris* is an emerging fungal pathogen with cases reported in countries around the world and in 19 states within the United States as of August 2020. The CDC has recommended that hospitals perform active surveillance upon admission for patients with the appropriate risk factors. Currently, active surveillance requires that local hospitals send surveillance swabs to a public health laboratory for analysis. In this work, a real-time PCR assay was developed for the specific detection of *C. auris* from surveillance swabs, blood, and urine to enable rapid detection of this pathogen. The assay uses commercially available primers and reporter probes and it was verified on the LightCycler 480 PCR platform. Contrived specimens and prospectively collected composite groin/axilla surveillance swabs were used to validate the assay. The performance of the PCR assay on surveillance swabs was also compared to a second PCR assay targeting *C. auris* that was performed at the Minnesota Department of Health–Public Health Laboratory (MDH-PHL). Our PCR assay is able to detect and differentiate *C. auris* from closely related *Candida* species such as *C. duobushaemulonii*, *C. haemulonii*, and *C. pseudohaemulonii* on the basis of melting curve temperature differences.

## 1. Introduction

*Candida auris* is a globally emerging, multidrug-resistant fungal pathogen with the potential to cause serious invasive infections [1,2,3,4,5]. Outbreaks of *C. auris* have occurred in healthcare settings across the globe, including in the U.S. regions of California, Florida, New Jersey, New York City, and Chicago, Illinois. These outbreaks have been difficult to control due to the ability of *C. auris* to contaminate the patient care environment and survive on surfaces for several weeks. Rapid, accurate identification and immediate implementation of infection control measures for patients infected or colonized with *C. auris* are crucial to controlling the spread.

Several *C. auris* cases have been linked to receipt of healthcare in countries outside the United States. The CDC recognizes foreign healthcare exposure as a risk factor for *C. auris*, and currently recommends that U.S. healthcare facilities identify the species of all *Candida* isolates from patients who had an overnight stay in a healthcare facility outside the U.S. in the previous year, especially for patients with stays at those facilities with documented *C. auris* transmission [6]. As of 8 May 2020, 1122 clinical cases of *C. auris* have been identified in 19 U.S. states, and an additional 2253 patients have been found with *C. auris* colonization in 16 jurisdictions [7]. Testing in these states has shown axilla and groin composite swabs to be the most common and consistent sites for finding *C. auris* colonization [6].

While the CDC recommends admission screening for carbapenemase-producing carbapenem-resistant enterobacteriaceae (CP-CRE) and *C. auris* among patients who received healthcare abroad in the past year, this recommendation has been challenging to implement for *C. auris* largely due to a lack of laboratory capacity across the U.S. for *C. auris* colonization testing. The CDC and Antibiotic Resistance (AR) Lab Network regional public health laboratories in the U.S. have PCR methods available to use for rapid screening of surveillance swabs [1,8,9]. The literature also contains a few reports of PCR assays at independent laboratories that were verified using culture isolates of *C. auris* or contrived specimens [10,11,12,13]. Currently, many hospital laboratories must send surveillance swabs to the CDC or one of the AR Lab Network Regional laboratories performing the PCR assay or they must rely on routine fungal culture for screening of surveillance swabs. Fungal culture requires two or more days to complete and supplementation of the culture medium with dulcitol may be needed to enhance recovery in colonized individuals. Following growth in culture, identification of *C. auris* to the genus and species level requires use of either matrix-assisted, laser desorption-time of flight (MALDI-TOF) mass spectrometry (MS) or gene sequencing [14,15]. Laboratories must use caution in selecting the MALDI-TOF MS library to use for identification since some may not contain *C. auris* [16].

The goal of this work was to design and verify a real-time PCR method for the specific detection of *C. auris* directly from surveillance swabs, blood, and urine. Blood and urine were selected because these specimens are the sources most frequently positive in clinical cases when isolates were submitted to our laboratory for identification by clinical laboratories across the country. A PCR method for the direct detection and identification of *C. auris* is desirable because of its potential to identify colonized or infected patients more rapidly than fungal culture. The PCR can be performed on the day of patient admission while fungal culture results for *Candida* species typically require two or more days. Rapid information about *C. auris* colonization is used at our institution to inform infection prevention and control practices. Colonized patients are placed in contact precautions to reduce the likelihood of spread to other patients or staff. In addition, the PCR method was designed to differentiate *C. auris* from other closely related *Candida* species. Phenotypic and semi-automated identification methods have been reported to misidentify *C. auris* as other fungi such as *C. duobushaemulonii*, *C. haemulonii*, and *C. psuedohaemulonii,* whereas the PCR method developed is specific for *C. auris*. In this report, the PCR assay is described and its performance from blood and urine was characterized using contrived specimens because of a lack of clinical specimens. The PCR assay performance on fresh, prospectively collected surveillance swabs was also compared with the *C. auris* real-time PCR assay performed at the Minnesota Department of Health–Public Health Laboratory (MDH-PHL).

## 2. Materials and Methods

### 2.1. Specimens

Negative blood and urine specimens were waste specimens remaining after routine clinical care processes were completed. Composite groin/axilla surveillance swabs were collected prospectively following consent of the patient. The use of waste specimens and the prospective collection of surveillance swabs was approved by an Institutional Review Board of the Mayo Clinic on 5/22/2019 (19-002552).

Patient inclusion criteria for the collection of *C. auris* surveillance swabs required the patient to be admitted to an inpatient unit. In addition, the patient had to reside in or have received healthcare in a part of the U.S. or in a country with documented *C. auris* cases within the past year or the patient was positive for CP-CRE (KPC, NDM, OXA-48, VIM) on clinical testing or surveillance testing [17]. Australia, Canada, China, Colombia, France, Germany, India, Israel, Japan, Kenya, Kuwait, Oman, Pakistan, Panama, Russia, Saudi Arabia, Singapore, South Africa, South Korea, Spain, United Kingdom, United States “hot spots,” and Venezuela were included at the time of study. United States “hot spots” included the states of CA, CT, TX, OH, RI, NY, NJ, VA, PA, NC, as well as Washington DC, Puerto Rico, and Chicago IL patients with a zip code of 60601, 60007, 60018, 60068, 60106, 60131, 60176, and 60686. Patients who had tested negative for *C. auris* in the past six months or who were <18 years old were excluded.

### 2.2. Culture Isolates

In order to test the PCR assay’s ability to detect various strains of *C. auris*, type strains of *Candida* species were obtained from the Deutsche Sammlung von Mikroorganismen (DSMZ; Braunschweig, Germany) and the American Type Culture Collection (ATCC; Masassas, VA, USA). In addition, a panel of *C. auris*, *C. haemulonii*, *C. duobushaemulonii,* and other yeast isolates was obtained from the CDC and FDA Antibiotic Resistance Isolate Bank (AR Bank Panel #1099 *C. auris*, Atlanta, GA, USA). Clinical isolates sent to our reference laboratory for identification were also tested by the PCR assay and the source of each isolate is indicated in Appendix A Table A1. *C. auris* was identified by MALDI-TOF MS using a Bruker BioTyper system and the BDAL library with 8468 MSPs and supplemented with a custom library containing an additional 2631 MSPs. Isolates were freshly sub-cultured onto Inhibitory Mold Agar (IMA) (BBL, Sparks, MD, USA). Following growth, culture isolates were lysed by placing a 1 µL loopful of organism into a 1 mL tube containing 500 µL of sterilized water, 0.1 mm silica glass beads, and 2.4 mm Zirconia beads (BioSpec Products Inc, Bartlesville, OK, USA). The tubes were heated at 95 °C for 10 min, and then placed on a Disruptor Genie (Scientific Industries Inc., Bohemia, NY, USA) for 2 min to mechanically lyse the organisms and release the nucleic acid. 5 µL of nucleic acid was placed into 15 µL of PCR master mix (described below) and was tested without further processing using the PCR assay.

### 2.3. Specimen Processing and Nucleic Acid Extraction

Whole blood containing EDTA preservative, urine, and composite axilla/groin swabs were used to validate the PCR assay. Owing to a lack of patient specimens containing *C. auris*, each specimen type was validated using contrived samples spiked with *C. auris* near the limit of detection of the PCR assay.

Whole blood (200 µL) containing EDTA as a preservative was extracted on the MagNA Pure LC 2.0 Instrument (Roche Diagnostics, Indianapolis, IN, USA) using the MagNA Pure LC Total Nucleic Acid Isolation Kit, with an elution volume of 100 µL.

Urine was concentrated to 5 mL by centrifugation if the volume received was >10 mL. 250 µL of urine was heated at 95 °C for 5 min and 200 µL was extracted on the MagNA Pure LC 2.0 Instrument using the MagNA Pure LC Total Nucleic Acid Isolation Kit, and a final elution volume of 100 µL.

Surveillance swab types tested were (1) soft aluminum-wire swabs with a rayon head in liquid Stuart medium (Catalog #220133, BD Diagnostics, Franklin Lakes, NJ, USA), (2) plastic shafted culture swabs with a rayon head in liquid Stuart medium (catalog #220099, BD Diagnostics), and (3) nylon flocked Eswab in liquid Amies medium (Catalog #220245, BD Diagnostics). The aluminum wire swab and the plastic shafted swab were processed by cutting the swab above the swab head and placing the swab head into a tube containing 600 µL of Tris-EDTA neutralization buffer (NB). The NB tube was then placed on a thermomixer and shaken at 14,000 rpm for 6 min at 100 °C. Eswabs were processed by placing 60 µL of the liquid in the Eswab transport container into an NB tube and shaking at 14,000 rpm for 6 min at 100 °C on a thermomixer.

### 2.4. PCR Assay Conditions

**Figure 1:**
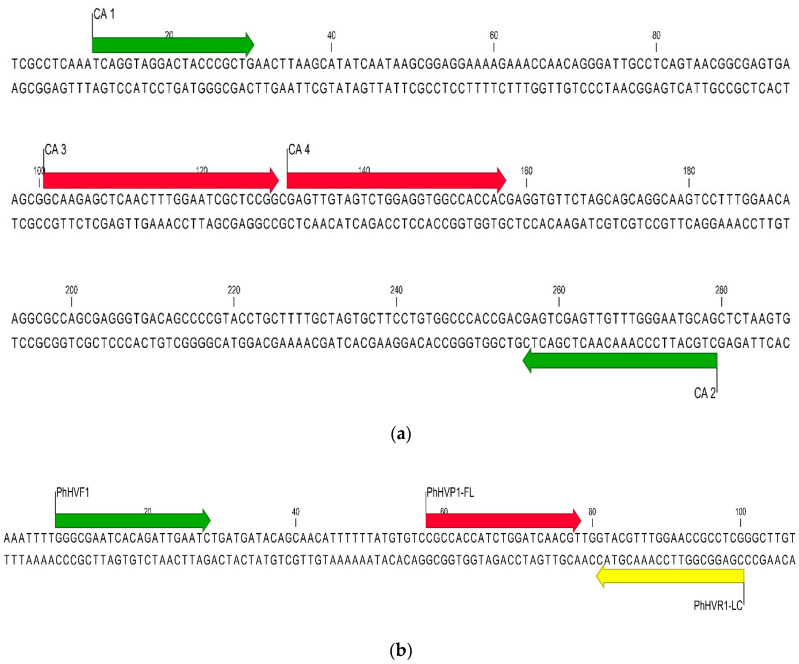
(**a**) Sequence alignment of the *C. auris* internal transcribed spacer (ITS) target region with the PCR assay primers (green arrows) and probes (red arrows). (**b**) Sequence alignment of the Phocine herpesvirus type 1 internal control target region with the PCR assay primers (green and yellow arrows) and probes (red and yellow arrows).

The real-time PCR assay was developed for use on the LightCycler 480 instrument (Roche Life Science, Madison, WI, USA). *C. auris* primers and probe sequences were designed to detect a 269 bp region of the internal transcribed spacer 2 (ITS2) of the ribosomal gene (Figure 1a). The donor probe is labeled with fluorescein and the acceptor probe with a LightCycler^®^ Red 610 nm fluorophore dye. The ITS target was chosen because of its highly conserved nature and the availability of sequence within public nucleotide databases [18].

Primers and probes were synthesized by TIB MOLBIOL (Adelphia, NJ, USA), and their sequences and product number are provided in Table 1. The PCR assay was performed using the LC FastStart DNA Master hybridization probe kit (Roche Diagnostics). Each reaction contained 0.8 µL of 25 mM MgCl_2_, 2 µL of 1× Roche LC FastStart mix, 0.03 µL of Recovery Template (PhHvµ DNA 10^6^), 0.5 µM of each forward primer, 1 µM of each reverse primer, 0.2 µM of fluorescein-labeled probe, and 0.4 µM of Red 610-labeled probe. The total volume per reaction was 20 µL (15 µL master mix plus 5 µL of nucleic acid). PCR amplification with real-time detection was performed using the following cycling parameters: 1 template denaturing cycle at 95 °C for 10 min, followed by 45 amplification cycles at 95 °C for 10 s, 55 °C for 15 s, and 72 °C for 20 s. Following amplification, melting curve analysis was performed by measuring the fluorescent signal during the following cycling parameters: 95 °C for 30 s, 59 °C for 10 s, 45 °C for 15 s with a 0.1 °C/s transition, and 85 °C for 0 s with a 0.1 °C/s transition.

### 2.5. PCR Assay Controls

A positive control plasmid containing the ITS2 target of *C. auris* was purchased from TIB MOLBIOL (TIB #4605). An internal control plasmid was also purchased from TIB MOLBIOL (TIB #30-8393-02). The plasmid contains the PhHV1 glycoprotein B gene from *Phoca vitulina*, a herpes virus that infects harbor seals. Primers and probes were designed to detect a 93 base pair region within the PhHV1 gene (Table 1 and Figure 1b). The donor probe is labeled with fluorescein while the acceptor probe is labeled with LightCycler^®^ Red 670 fluorophore.

The negative control for extracted samples (blood, urine) consists of *Escherichia coli* ATCC #25922 in 50% Stool Transport and Recovery (S.T.A.R.) buffer (Product #03335208001, Roche Diagnostics). A sterile culture swab clipped into an NB tube is used for the surveillance swab negative control.

### 2.6. PCR Assay Limit of Detection

The limit of detection (LOD) for the PCR assay was determined for the blood, urine, and surveillance swabs. A 0.5 McFarland solution of *C. auris* was spiked into negative specimens at concentrations ranging from 1 colony forming unit (CFU) to 733 CFU. The CFU of each dilution was confirmed by plating the inoculum onto SAB plates and enumerating the colonies following growth at 30 °C. 200 µL of each dilution was extracted three times using the MagNA Pure 2.0 extraction system as described previously. Each DNA extract was tested in duplicate for a total of six PCR replicates per dilution. The limit of detection was defined as the concentration that was positive by the PCR assay in 6 out of 6 replicates. Positive and negative extraction controls were also included for quality assurance.

### 2.7. PCR Assay Precision

Intra-day assay precision was tested by spiking negative whole blood specimens with three concentrations (low ~50 CFU/reaction, intermediate ~200 CFU/reaction, high ~550 CFU/reaction) of *C. auris* suspended in sterile water. 200 µL of each spiked specimen was extracted on the MagNA Pure 2.0 instrument and was tested in triplicate with the PCR assay on the LC 480 instrument. All 9 replicates were tested on the same day.

Inter-day assay precision was tested by spiking negative whole blood with the same three concentrations (low, intermediate, high) of *C. auris* suspended in sterile water. On 3 separate days and using 3 different laboratory technologists, 200 µL of each of the spiked specimens were extracted on the MagNA Pure 2.0 instrument and tested in triplicate with the PCR assay on the LC 480 instrument (*n* = 9 replicates per day, *n* = 27 replicates total).

### 2.8. PCR Assay Accuracy

Thirty negative specimens for each specimen type (surveillance swabs, blood, urine) were spiked with *C. auris* at a concentration within 1 log of the LOD determined for that specimen type. 6 different clinical isolates of *C. auris* were used for spiking each type of specimen (i.e., 5 specimens per isolate) to examine the effect, if any, of different *C. auris* isolate strains on detection by the PCR assay. Specimens were processed as described previously and tested by the PCR assay.

### 2.9. PCR Assay Specificity

The analytical specificity of the *C. auris* PCR assay was examined in silico by performing a BLAST search of each primer, each probe, and the entire amplicon sequence using the National Center for Biotechnology Information (NCBI) GenBank BLAST search website [18]. 50 organisms from the NCBI database that were closest in sequence homology to the target region of the *C. auris* PCR assay were identified to predict potential cross-reactivity with the PCR assay. An additional 9 unrelated but common organisms were analyzed in silico (*Debaryomyces hansenii*, *Candida albicans*, *Candida glabrata*, *Candida tropicalis*, *Candida parapsilosis*, *Candida krusei*, *Aspergillus fumigatus*, *Penicillium* sp., and *Fusarium* sp.).

A panel of genomic DNA from 72 bacterial, fungal, and viral organisms found on skin, in blood, in urine, and in the environment were tested using the PCR assay for potential cross-reactivity with the primers and probes. Amplification and Sanger dideoxy sequencing of either 16S (bacteria), D2 LSU (fungi) rDNA, or viral-specific PCR assays [19,20] were utilized to confirm the presence of amplifiable nucleic acids in the specificity panel.

### 2.10. PCR Assay Comparison with MDH-PHL PCR Assay

The performance of the laboratory-developed PCR assay on surveillance swabs was compared with a real-time PCR assay performed by the MDH-PHL, the AR Lab Network Central Region laboratory. The assay was developed at the MDH-PHL (modifications to a previously described assay) [1]. Surveillance swabs were collected in duplicate from each consenting patient who met the CDC surveillance criteria. One swab was tested by the laboratory-developed PCR assay while the second swab was tested using the real-time PCR assay at the MDH-PHL. The swabs were stored and shipped at 4–25 °C and were tested within 4 days of collection.

### 2.11. Stability Studies

PCR master mix stability was tested up to 35 days of storage at 2 to 8 °C and −15 to −25 °C using the positive control plasmid. Specimen stability in each matrix type (blood, urine, surveillance swabs) was tested by spiking each matrix with 250 CFU/µl of *C. auris* and storing in individual aliquots at 2 to 8 °C and −15 to −25 °C. Individual aliquots were tested in triplicate after 0, 1, 8, and 14 days of storage. Extracted nucleic acid stability was tested by spiking negative extracts with 250 CFU/µL of *C. auris* and storing in individual aliquots at 2 to 8 °C and −15 to −25 °C. Individual aliquots were tested in triplicate after 0, 1, 8, and 14 days of storage.

## 3. Results

### 3.1. Detection of C. auris Culture Isolates

**Figure 2:**
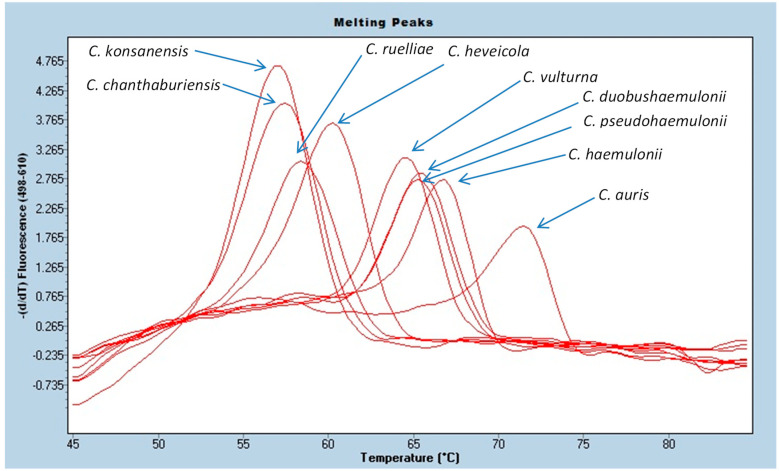
PCR melting curves for *C. auris* and closely related *Candida* species.

The PCR assay detected 32 of 32 (100%) culture isolates of *C. auris* (Appendix A Table A1) with an average Cp of 19.12 ± 1.94 cycles and an average melting temperature of 70.59 ± 0.13 °C. A representative melting curve is shown in Figure 2.

All of the *C. auris* isolates in the CDC and FDA Antibiotic Resistance Isolate Bank panel (#1099) as of 5/22/2020 were detected by the PCR assay (*n* = 12). *C. haemulonii* (*n* = 1) and *C. duobushaemulonii* (*n* = 2) were also detected by the PCR but were differentiated from *C. auris* on the basis of melting temperature differences. Other yeast isolates in the panel were not detected by PCR assay.

### 3.2. PCR Assay Limit of Detection

The LOD of the PCR assay using *C. auris* spiked into EDTA whole blood samples was determined to be 54 CFU/reaction. The LOD in urine was 37 CFU/reaction. The LOD in aluminum-shafted rayon, plastic-shafted rayon, and Eswabs was 4, 11, and 37 CFU/reaction, respectively.

### 3.3. PCR Assay Precision

The precision results are presented in Appendix A Table A2 and Table A3. For intra-day precision, all 9 replicates were detected (100%). The average crossing point (Cp) was 27.79 ± 0.18 cycles at the high concentration (550 CFU/reaction), 28.54 ± 0.35 cycles at the intermediate concentration (200 CFU/reaction), and 35.22 ± 0.99 cycles at the low concentration (50 CFU/reaction). All Cps for the 9 replicates were within ± 2 cycles of the average. The average Tm was 69.99 ± 0.06 °C for the high concentration, 70.24 ± 0.26 °C for the intermediate concentration, and 70.47 ± 0.12 °C for the low concentration.

For inter-day precision, all 27 replicates were detected (100%). The average Cp was 29.15 ± 0.86 cycles at the high concentration, 30.47 ± 0.78 cycles at the intermediate concentration, and 33.08 ± 0.82 cycles at the low concentration. The average Tm was 70.68 ± 0.20 °C for the high concentration, 70.69 ± 0.20 °C for the intermediate concentration, and 70.96 ± 0.32 °C for the low concentration. No differences in results between days or between technologists were noted.

### 3.4. PCR Assay Accuracy

The PCR assay detected *C. auris* in each specimen matrix type with an accuracy of ≥93.3% (Table 2). The assay detected 29/30 whole blood specimens (96.7%), 29/30 urine specimens (96.7%), 30/30 aluminum-shafted rayon swab specimens (100%), 28/30 plastic-shafted rayon swab specimens (93.3%), and 28/30 Eswab specimens (93.3%). One of the six specimens that was not detected, a plastic-shafted rayon swab, was inhibited, as demonstrated by a negative internal control. Each specimen type had an amplification curve standard deviation of ≤2.56 cycles and a melting temperature standard deviation of ≤0.39 °C for the 30 contrived specimens.

The agreement of the laboratory-developed PCR assay with the MDH-PHL PCR assay was 100% (Table 3). Sixty-five surveillance swab specimens were negative by both PCR assays and one swab was inhibited in both PCR assays.

### 3.5. PCR Assay Specificity

**Figure 3:**
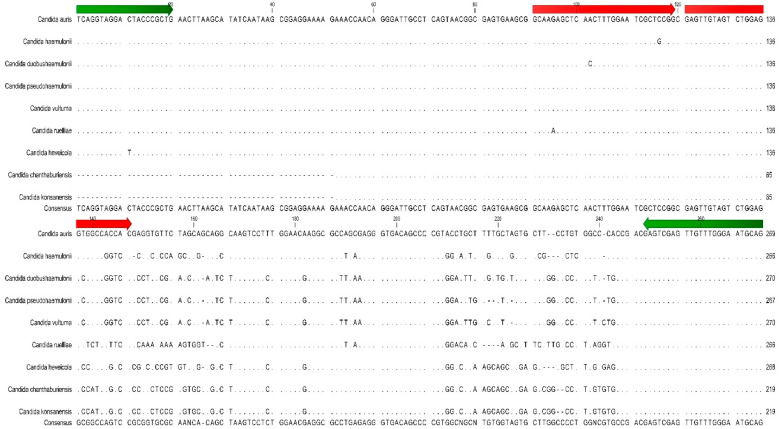
Sequence alignment of *C. auris* ITS target region with 8 closely related Candida species. Green arrows indicate the position of the PCR assay primers. Red arrows indicate the position of the PCR assay reporter probes.

The laboratory-developed PCR was found to be highly specific for *C. auris*. As expected, none of the bacteria, fungi, or viruses tested were positive in the PCR assay with the exception of the closely related *Candida* species such as *C. duobushaemulonii*, *C. haemulonii*, and *C. pseudohaemulonii* (Appendix A Table A4). These 3 species can be misidentified as *C. auris* using some identification systems. While they are detected by the laboratory-developed PCR assay, their melting peak temperatures (Tm = 65.09–66.50 °C) are sufficiently different from *C. auris* (Tm = 70.59 °C) to readily allow differentiation from *C. auris* based on melting temperature differences. The melting temperatures of *C. duobushaemulonii*, *C. haemulonii,* and *C. pseudohaemulonii* are within 1.5 °C of each other so they cannot be differentiated from each other by melting curve analysis, but they are distinct from *C. auris* (Figure 2). Other closely related but infrequently isolated *Candida* species (i.e., *Candida chanthaburiensis*, *Candida heveicola*, *Candida konsanensis*, *Candida ruelliae*, *Candida vulturna*) also produced melting curves that overlapped each other but that were distinct from *C. auris* by several degrees (Figure 2). Using an in silico analysis of 60 organisms in GeneBank with the closest ITS2 sequence homology did not identify any homology in the probe binding region that would cause concern for cross-reactivity (Appendix A Table A4 and Figure 3).

### 3.6. Stability Studies

The PCR master mix was stable at 2 to 8 °C and −15 to −25 °C up to 35 days. Specimen stability and extract stability varied by specimen type. EDTA whole blood, urine, aluminum-shafted rayon swabs, and Eswab specimens and nucleic acid extracts were stable after storage at 2 to 8 °C and −15 to −25 °C for 14 days. Plastic-shafted rayon swabs specimen stability was only good for 1 day at both 2 to 8 °C and −15 to −25 °C, so that swab type must be tested on the day collected to avoid loss of sensitivity of the assay and the other swabs types are preferred due to better specimen stability over time.

## 4. Discussion

The laboratory-developed PCR assay was found to have good sensitivity from blood, urine, and a variety of surveillance swab types. The Eswab is the preferred type for surveillance due to its good sensitivity, extended specimen stability, and its simple processing requirements. The flocked Eswab releases the specimen into the liquid of the swab container, allowing laboratory staff to sample the liquid without the requirement to cut the swab head off first into the liquid. This is ergonomically preferred for laboratory staff who can avoid potential repetitive motion injuries associated with clipping of swabs, and it also reduces the potential for contamination of the laboratory work area with target nucleic acid during the cutting process.

The sensitivity of the assay from blood and urine using contrived specimens was ≤100 CFU/reaction, so the PCR assay can be useful for the direct detection of *C. auris* from these specimen types. The PCR test should be ordered on blood and urine specimens only from those patients who are strongly suspected to have *C. auris* disseminated infection based on a review of symptoms and risk factors, such as recent foreign hospitalization [6]. Clinicians must not order the PCR assay on blood or urine for surveillance of colonization or for suspected infections at other local sites (e.g., respiratory, wound) because the sensitivity of these specimen types to detect colonization is unproven at this time. Although these specimens are easily obtained, especially urine, these are not appropriate or optimal specimen types for the detection of colonization or localized infections. Collection of composite axilla/groin surveillance swabs is encouraged for surveillance purposes.

The PCR assay detects *C. auris* isolates that belong to each of the currently recognized clades. In silico analysis of the target sequence and the primers and probes utilized in the PCR assay predicted that the assay would detect all clades. Testing of the CDC and FDA Antibiotic Resistance Isolate Bank panel, which contains *C. auris* strains from all clades (East Asia (*n* = 1), South Asia (*n* = 5), Africa (*n* = 2), South America (*n* = 3), and Iran (*n* = 1)), confirmed that the PCR assay detects all clades identified to date.

The ability to rapidly and directly detect *C. auris* from surveillance swabs and specimens such as blood and urine is important to provide another diagnostic tool to assist with containing the spread of this emerging fungal pathogen. The MDH-PHL is one of seven labs in CDC’s AR Lab Network to receive funding support for enhanced capacity to detect and respond to emerging antimicrobial resistance threats, including *C. auris*. One tool the AR Lab Network labs use to stop the spread of *C. auris* is colonization testing, which has been implemented as targeted screening in response to clinical cases, as well as for admission screening for specifically defined patients.

To date, *C. auris* has been identified in one patient in Minnesota who travelled and received healthcare outside of the U.S. MDH-PHL and epidemiologists continue to work with the CDC, as well as local and regional healthcare facilities to ensure that the state is prepared to identify and respond to cases of *C. auris*. As part of these efforts, the MDH-PHL AR Lab Network laboratory and MDH epidemiologists worked with the Mayo Clinic to implement *C. auris* colonization screening among patients admitted from foreign countries and U.S. regions with ongoing *C. auris* transmission because the Mayo Clinic is more likely than many hospitals in the state to admit patients with *C. auris* colonization. While the MDH-PHL/Mayo Clinic partnership works well, there is also a need for healthcare facilities to have the ability to perform *C. auris* screening on site. Testing of surveillance swabs at a centralized public health lab requires local, trained resources at healthcare facilities for packaging and shipping of specimens. This results in delayed testing of the surveillance swabs by at least one day and potentially longer depending on local hospital resources and the need to batch swabs prior to shipping. In addition, public health resources are not limitless, so having alternative avenues for testing and surveillance can be useful when public health resources are strained. For those healthcare facilities with the need and the capacity, the laboratory-developed PCR assay described in this work can provide a method that can be implemented locally following verification. The commercial availability of the primers and probes and description of assay conditions in this work can assist with local adoption.

## Figures and Tables

**Table 1:** *Candida auris* PCR assay primer and probe sequences.

Primer/Probe	Abbreviation	TIB MOLBIOL Number	Nucleotide Sequence (5′-3′)
**ITS Target**			
Forward primer	CA1	4605	5′ TCA GGT AGG ACT ACC CGC TG 3′
Reverse primer	CA2	4605	5′ CTG CAT TCC CAA ACA ACT CGA CTC 3′
Fluorescein-labeled probe	CA3	4605	5′ GCA AGA GCT CAA CTT TGG AAT CGC TCC GG -FL 3′
Red 610-labeled probe	CA4	4605	5′ LC610- GAG TTG TAG TCT GGA GGT GGC CAC CAC -P 3′
**PhHV Internal Control**			
Forward primer	PhHVF1	30-8393-02	5′ GGG CGA ATC ACA GAT TGA ATC 3′
Fluorescein-labeled probe	PhHVP1-FL	30-8393-02	5′ CGC CAC CAT CTG GAT CAA CGT -FL 3′
Red 670-labeled probe and Reverse primer	PhHVR1-LC	30-8393-02	5′ LC670- CGA GGC GGT TCC AAA CGX TAC -PH 3′

**Table 2:** Summary Cp and Tm (°C) results for contrived specimens spiked with *C. auris*.

Specimen Type	Concentration	No. Pos./No. Tested	% Positive	Mean Cp cycle (SD)	Mean Tm °C (SD)
Blood, whole w/EDTA	100 CFU/Rxn	29/30	96.7	33.37 (2.56)	71.12 (0.32)
Urine	75 CFU/Rxn	29/30	96.7	31.85 (2.01)	71.06 (0.25)
NP Swabs	15 CFU/Rxn	30/30	100	35.06 (1.68)	70.87 (0.31)
Culturette Swabs	30 CFU/Rxn	28/30	93.3	35.25 (1.56)	71.03 (0.34)
Eswabs	200 CFU/Rxn	28/30	93.3	34.76 (1.97)	70.69 (0.39)

CFU = colony forming units; Rxn = reaction.

**Table 3:** Comparison of the laboratory-developed PCR assay with the real-time PCR assay performed by the MDH-PHL.

		MDH-PHL PCR Result		
		Positive	Negative	Inhibited
Laboratory-developed PCR result	Positive	0	0	0
	Negative	0	65	0
	Inhibited	0	0	1
	Total	0	65	1

## Appendix A

**Table A1:** *Candida auris* isolates used to evaluate the PCR test.

Isolate ID	Organism	Source of Isolate	Geographic Origin	PCR Amplification Cp (Cycles)	PCR Melting Temperature Tm (°C)
CA1	*Candida auris*	Blood	Illinois	21.69	70.51
CA2	*Candida auris*	Urine	Illinois	20.14	70.46
CA3	*Candida auris*	Blood	Illinois	21.57	70.41
CA4	*Candida auris*	Blood	New Jersey	19.75	70.44
CA5	*Candida auris*	Axilla Wound	New Jersey	18.88	70.47
CA6	*Candida auris*	Groin Wound	New Jersey	19.30	70.48
CA7	*Candida auris*	Blood	New Jersey	19.72	70.44
CA8	*Candida auris*	Blood	New Jersey	19.90	70.34
CA9	*Candida auris*	Paranasal Sinus	New Jersey	19.78	70.50
CA10	*Candida auris*	Peritoneal Fluid	Massachusetts	17.04	70.58
CA11	*Candida auris*	Blood	Massachusetts	17.78	70.49
CA12	*Candida auris*	Catheter Tip PICC line	New Jersey	20.68	70.50
CA13	*Candida auris*	Urine	New Jersey	19.17	70.75
CA14	*Candida auris*	Perirectal	Massachusetts	18.41	70.76
CA15	*Candida auris*	Pleural Fluid	Saudi Arabia	20.88	70.66
CA16	*Candida auris*	Blood	New Jersey	21.68	70.52
CA17	*Candida auris*	Blood	Saudi Arabia	19.80	70.58
CA18	*Candida auris*	Blood	Saudi Arabia	18.28	70.48
CA19	*Candida auris*	Urine	Saudi Arabia	18.74	70.67
CA20	*Candida auris*	Blood	New Jersey	22.23	70.55
CA21	*Candida auris*	Axilla/Groin Swab	New Jersey	22.88	70.56
CA22	*Candida auris*	Urine	New Jersey	21.40	70.69
CA23	*Candida auris*	Axilla/Groin Swab	New Jersey	20.74	70.53
CA24	*Candida auris*	Axilla/Groin Swab	New Jersey	15.91	70.91
CA25	*Candida auris*	Urine	New Jersey	16.22	70.67
CA26	*Candida auris*	Axilla/Groin Swab	New Jersey	16.81	70.70
CA27	*Candida auris*	Sacrum Ulcer Swab	Saudi Arabia	17.33	70.75
CA28	*Candida auris*	Axilla/Groin Swab	New Jersey	16.51	70.72
CA29	*Candida auris*	Abdominal Wound	New Jersey	16.94	70.72
CA30	*Candida auris*	Blood	New Jersey	16.79	70.74
CA31	*Candida auris*	Urine	Saudi Arabia	17.66	70.71
CA32	*Candida auris*	Urine	New Jersey	17.35	70.72
Average Range—Min Range—Max SD				19.12	70.59
				15.91	70.34
				22.88	70.91
				1.94	0.13

**Table A2:** Intra-day precision: 3 replicates tested at 3 concentrations (high, medium, low) on the same day.

Replicate #	High Concentration (550 CFU/Rxn)		Medium Concentration (200 CFU/Rxn)		Low Concentration (50 CFU/Rxn)	
	Cp (cycles)	Tm (°C)	Cp (cycles)	Tm (°C)	Cp (cycles)	Tm (°C)
1	27.83	70.02				
2	27.60	69.92				
3	27.95	70.02				
4			28.83	70.02		
5			28.15	70.52		
6			28.63	70.17		
7					35.35	70.59
8					34.18	70.35
9					36.14	70.46
Average	27.79	69.99	28.54	70.24	35.22	70.47
Range—Min	27.60	69.92	28.15	70.02	34.18	70.35
Range—Max	27.95	70.02	28.63	70.52	36.14	70.59
SD	0.18	0.06	0.35	0.26	0.99	0.12

CFU = colony forming unit; Rxn = reaction.

**Table A3:** Inter-day precision: 3 replicates tested at 3 concentrations (high, medium, low) over 3 days, with testing performed by 3 technologists.

	**High Concentration (550 CFU/Rxn)**		
**Date Assayed**	**Replicate #**	**Cp (cycles)**	**Tm (°C)**
Day 1 Tech: 1	1	30.06	70.90
	2	30.30	71.05
	3	29.37	70.59
Day 2 Tech: 2	4	29.08	70.40
	5	29.34	70.47
	6	29.75	70.66
Day 3 Tech: 3	7	28.48	70.70
	8	28.24	70.70
	9	27.70	70.65
Average		29.15	70.68
Range—Min		27.70	70.40
Range—Max		30.30	71.05
SD		0.86	0.20
	**Medium Concentration (200 CFU/Rxn)**		
**Date Assayed**	**Replicate #**	**Cp (cycles)**	**Tm (°C)**
Day 1 Tech: 1	1	31.11	70.90
	2	31.52	71.05
	3	30.83	70.59
Day 2 Tech: 2	4	30.65	70.63
	5	30.43	70.40
	6	30.96	70.48
Day 3 Tech: 3	7	30.11	70.75
	8	29.10	70.75
	9	29.48	70.70
Average		30.47	70.69
Range—Min		29.10	70.40
Range—Max		31.52	71.05
SD		0.78	0.20
	**Low Concentration (50 CFU/Rxn)**		
**Date Assayed**	**Replicate #**	**Cp (cycles)**	**Tm (°C)**
Day 1 Tech: 1	1	33.82	71.04
	2	34.24	71.10
	3	33.20	70.91
Day 2 Tech: 2	4	32.94	70.24
	5	31.76	70.77
	6	33.34	70.90
Day 3 Tech: 3	7	32.54	71.25
	8	33.76	71.24
	9	32.12	71.20
Average		33.08	70.96
Range—Min		31.76	70.24
Range—Max		34.24	71.25
SD		0.82	0.32

CFU = colony forming unit; Rxn = reaction.

**Table A4:** Specificity panel of organisms tested by the PCR assay.

Organism	Source	PCR Assay Result	Melting Peak Tm (°C)	Genomes/µL
**Bacteria**				
*Acinetobacter baumannii*	ATCC 19606	Negative	ND	2.01 × 10^11^
*Bacillus subtilis* group	Patient isolate	Negative	ND	6.83 × 10^7^
*Borrelia burgdorferi*	ATCC 35210	Negative	ND	2.06 × 10^10^
*Bartonella henselae*	ATCC 25583	Negative	ND	1.10 × 10^8^
*Clostridium difficile*	ATCC 9689d-5	Negative	ND	4.69 × 10^9^
*Corynebacterium amycolatum*	Patient isolate	Negative	ND	3.41 × 10^8^
*Corynebacterium aurimucosum/minutissimum*	Patient isolate	Negative	ND	1.65 × 10^8^
*Corynebacterium striatum*	Patient isolate	Negative	ND	5.23 × 10^7^
*Cutibacterium acnes*	ATCC 6919	Negative	ND	6.91 × 10^7^
*Enterobacter cloacae*	ATCC 13047	Negative	ND	5.51 × 10^8^
*Enterococcus faecalis*	ATCC 19433-U	Negative	ND	3.41 × 10^9^
*Enterococcus faecium*	ATCC 19434	Negative	ND	4.67 × 10^9^
*Escherichia coli*	ATCC 25922	Negative	ND	1.33 × 10^10^
*Haemophilus influenzae*	ATCC 10211	Negative	ND	4.36 × 10^9^
*Klebsiella pneumoniae*	ATCC 700603	Negative	ND	3.41 × 10^10^
*Legionella pneumophila*	ATCC 33152	Negative	ND	6.31 × 10^9^
*Listeria monocytogenes*	ATCC 15313	Negative	ND	1.63 × 10^9^
*Mycobacterium avium*	ATCC 700398	Negative	ND	4.15 × 10^7^
*Mycobacterium haemophilum*	ATCC 29548	Negative	ND	5.47 × 10^7^
*Mycoplasma pneumoniae*	ATCC 15531	Negative	ND	4.00 × 10^8^
*Pseudomonas aeruginosa*	ATCC 27853	Negative	ND	1.55 × 10^10^
*Staphylococcus aureus*	ATCC 25923	Negative	ND	4.24 × 10^9^
*Staphylococcus epidermidis*	ATCC 14990	Negative	ND	1.42 × 10^9^
*Streptococcus mitis* group	Patient isolate	Negative	ND	1.29 × 10^8^
*Streptococcus pyogenes*	ATCC 19615	Negative	ND	1.21 × 10^9^
*Tropheryma whipplei*	Patient blood	Negative	ND	6.41 × 10^9^
**Filamentous Fungi**				
*Alternaria* species	Patient isolate	Negative	ND	2.72 × 10^7^
*Aspergillus flavus*	ATCC 204304	Negative	ND	1.58 × 10^7^
*Aspergillus fumigatus*	ATCC MYA-3626	Negative	ND	4.83 × 10^7^
*Blastomyces dermatitidis*	Patient isolate	Negative	ND	2.27 × 10^6^
*Curvularia pallescens*	ATCC 60941	Negative	ND	2.77 × 10^7^
*Emergomyces africanus*	Patient isolate	Negative	ND	1.15 × 10^7^
*Epidermophyton floccosum*	CBS 970.25	Negative	ND	4.04 × 10^7^
*Fusarium* species	NIH-54	Negative	ND	3.91 × 10^7^
*Histoplasma capsulatum*	Patient isolate	Negative	ND	1.47 × 10^7^
*Microsporum canis*	NIH-25	Negative	ND	3.37 × 10^7^
*Paecilomyces* species	NIH-56	Negative	ND	1.99 × 10^7^
*Penicillium* species	NIH-34	Negative	ND	3.95 × 10^7^
*Rhizopus* species	DSMZ-1834	Negative	ND	7.02 × 10^6^
*Scopulariopsis* species	NIH-49	Negative	ND	2.80 × 10^7^
*Trichophyton rubrum*	ATCC MYA-4438	Negative	ND	3.65 × 10^7^
**Yeast**				
*Candida albicans*	ATCC 60193	Negative	ND	5.70 × 10^8^
*Candida duobushaemulonii*	CBS 7798	Positive	65.09	3.92 × 10^6^
*Candida glabrata*	ATCC 15126	Negative	ND	1.20 × 10^7^
*Candida haemulonii*	ATCC 22991	Positive	66.50	8.57 × 10^6^
*Candida intermedia*	ATCC 14439	Negative	ND	3.21 × 10^6^
*Candida krusei*	ATCC 6258	Negative	ND	3.20 × 10^6^
*Candida parapsilosis*	ATCC 22019	Negative	ND	2.58 × 10^6^
*Candida pseudohaemulonii*	CBS 12370	Positive	65.13	5.16 × 10^6^
*Candida pseudointermedia*	ATCC 60126	Negative	ND	3.19 × 10^6^
*Candida tropicalis*	ATCC 1369	Negative	ND	1.53×10^6^
*Clavispora lusitaniae*	Patient isolate	Negative	ND	3.25 × 10^6^
*Cryptococcus gattii*	ATCC 32269	Negative	ND	1.66 × 10^8^
*Cryptococcus neoformans*	ATCC 28957	Negative	ND	4.85 × 10^7^
*Debaryomyces hansenii*	Patient isolate	Negative	ND	9.64 × 10^6^
*Malassezia furfur*	ATCC 12078	Negative	ND	1.31 × 10^7^
*Pneumocystis* species	Patient isolate	Negative	ND	4.54 × 10^6^
*Rhodotorula mucilaginosa*	Patient isolate	Negative	ND	1.44 × 10^7^
*Saccharomyces cerevisiae*	ATCC 4126	Negative	ND	5.42 × 10^6^
*Trichosporon cutaneum*	ATCC 28592	Negative	ND	1.26 × 10^7^
*Trichosporon dermatis*	ATCC MYA-4294	Negative	ND	1.59 × 10^7^
**Parasite/Virus/Human**				
*Acanthamoeba polyphaga*	ATCC 50371	Negative	ND	1.00 × 10^5^
*Balamuthia mandrillaris*	ATCC PRA290	Negative	ND	1.00 × 10^3^
*Cyclospora* species	Patient stool	Negative	ND	6.96 × 10^5^
*Dientamoeba fragilis*	ATCC 30948	Negative	ND	2.74 × 10^5^
*Giardia lamblia*	ATCC 30957	Negative	ND	3.21 × 10^8^
*Naegleria fowleri*	ATCC 30896	Negative	ND	1.00 × 10^5^
*Plasmodium falciparum*	WHO STD	Negative	ND	1.00 × 10^6^
Cytomegalovirus	AcroMetrix	Negative	ND	5.00 × 10^8^
Epstein-Barr virus	AcroMetrix	Negative	ND	5.00 × 10^8^
Human DNA	MRC-5 cells	Negative	ND	3.42 × 10^7^

AcroMetrix = ThermoFisher AcroMetrix controls, Waltham, MA; ATCC = American Type Culture Collection, Manassas, VA; CBS = CBS-KNAW Collections, Westerdijk Fungal Biodiversity Institute, Utrecht, The Netherlands; DSMZ = Leibniz Institute DSMZ—German Collection of Microorganisms and Cell Cultures GmbH, Germany; NIH = National Institutes of Health, Bethesda, MD; ND = not detected; WHO = World Health Organization.
